# KLF15 promotes the proliferation and metastasis of lung adenocarcinoma cells and has potential as a cancer prognostic marker

**DOI:** 10.18632/oncotarget.21972

**Published:** 2017-10-19

**Authors:** Lihua Gao, Hongmei Qiu, Jian Liu, Yuzhen Ma, Jia Feng, Li Qian, Jianguo Zhang, Yifei Liu, Tingting Bian

**Affiliations:** ^1^ Department of Oncology, Affiliated Hospital of Nantong University, Nantong 226001, Jiangsu, China; ^2^ Department of Respiration, Nantong Geriatric Rehabilitation Hospital, Branch of Affiliated Hospital of Nantong University, Nantong 226001, Jiangsu, China; ^3^ Centre of Reproductive Medicine, Inner Mongolia Hospital, Inner Mongolia, Hohhot, 010021, China; ^4^ Department of Pathology, Affiliated Hospital of Nantong University, Nantong 226001, Jiangsu, China

**Keywords:** KLF15, lung adenocarcinoma, cell proliferation, cell migration, prognosis

## Abstract

Lung adenocarcinoma (LADC)is a general form of non-small cell lung cancer that represents a significant threat to public health worldwide. The 5-year-survival rate for LADC is currently below 15%. The transcription factor KLF15, also called kidney-enriched KLF (KKLF), has been proven to play a role in inhibiting proliferation and diversification of carcinoma cells, including those of the endometrium, pancreas and breast, but the involvement of KLF15 in LADC has not previously been studied. In this study, we compared the *in vitro* expression of KLF15 in human LADC tissues and adjacent normal lung tissues. Expression of KLF15 was found to be abnormally high in LADC tissues and cells compared with adjacent non-tumorous tissues, and was correlated with tumor TNM stage and tumor differentiation (*P* = 0.003, *P* = 0.001, respectively). The effect of KLF15 on cell growth and migration were explored *in vitro* by Western Blotting, CCK8 and colony formation assays, flow cytometry analysis and transwell migration assays, and *in vivo* by analysis of tumorigenesis in 5-week old BALB/c nude mice. Knockdown of KLF15 significantly upregulated the protein levels of cleaved caspase-3, caspase-7, caspase-8 and PARP, thereby inducing apoptosis. Downregulation of KLF15 in A549 and NCI-H1650 cell lines resulted in these cell lines exhibiting markedly slower growth rates when injected subcutaneously into the flank of nude mice, compared with the comparator control groups (*P* < 0.05). Collectively, our findings suggest that KLF15 may have a significant effect on LADC cell survival, and that it represents a potential therapeutic and preventive biomarker for LADC prognosis and treatment.

## INTRODUCTION

Lung cancer is currently the leading cause of cancer-related deaths worldwide. Lung adenocarcinoma (LADC) is the most common histological type of lung cancer and has increased in incidence in recent years, now accounting for more than 500,000 deaths per year worldwide [1, 2]. It is known that lung cancer biology is complex and influenced by multiple genome aberrations and by aberrant activation of proteins involved in cell signaling pathways. Ongoing clinical and laboratory research has predominantly focused on the molecular pathways involved in the tumorigenesis and progression of lung cancer. The studies conducted to date suggest that bone morphogenetic proteins (BMPs) [3], homeodomain-containing transcription factors (such as HOXB9) [4], and Notch3 signaling pathways [5], are tumor-promoting in LADC, while forehead box protein O3 (FOXO3) is a suppressor of lung cancer carcinogenesis [6, 7]. Despite these advances in understanding, 5-year-survival rates for most patients diagnosed at the advanced stages of the disease are still less than 15% [8–10]. As a result, there is an urgent need to identify promising prognostic biomarkers and key molecules associated with the development and metastases of LADC.

The Krüppel-like factors (KLFs) make up a group of transcription factors in which the carboxy-terminal regions contain three different C_2_H_2_-type zinc finger domains. KLFs have a significant effect on both nuclear localization and DNA binding and individual proteins within the KLF family share common mechanisms of transcription regulation [11, 12]. To date, a few studies have demonstrated that several KLF family members regulate genes responsible for a diverse range of biological functions, for example, cellular proliferation, differentiation and apoptosis. The biological importance of KLF family proteins has been shown not only in the regulation of normal biological processes such as the maintenance of homeostasis, but also in the regulation of pathological conditions [11, 13]. In particular, KLF15 has been reported to be expressed in multiple tissues including the liver, kidney, adipose, heart, and skeletal muscle [14, 15]. However, its involvement in human malignancies has rarely been studied. Recent *in vitro* studies have found that KLF15 may have anti-proliferative effects on carcinoma cells in a range of cancers, including those of the endometrium, pancreas, and breast [16, 17].

In this study, we sought to investigate and compare the expression of KLF15 in human LADC tissues and adjacent normal lung tissues and to conduct a series of *in vitro* experiments including immunohistochemistry assays, transfection and knockdown experiments, cell proliferation and colony formation assays, to investigate the role of KLF15 in the pathogenesis of LADC. We also analyzed the *in vivo* effect of KLF15 on tumorigenicity in nude mice. It was anticipated that our findings would inform understanding about the role of KLF15 in LADC and the molecular mechanisms involved. This would potentially inform the future development of prognostic and treatment therapies for LADC.

## RESULTS

### Clinical significance of KLF15 expression in LADC tissues

**Figure 1:**
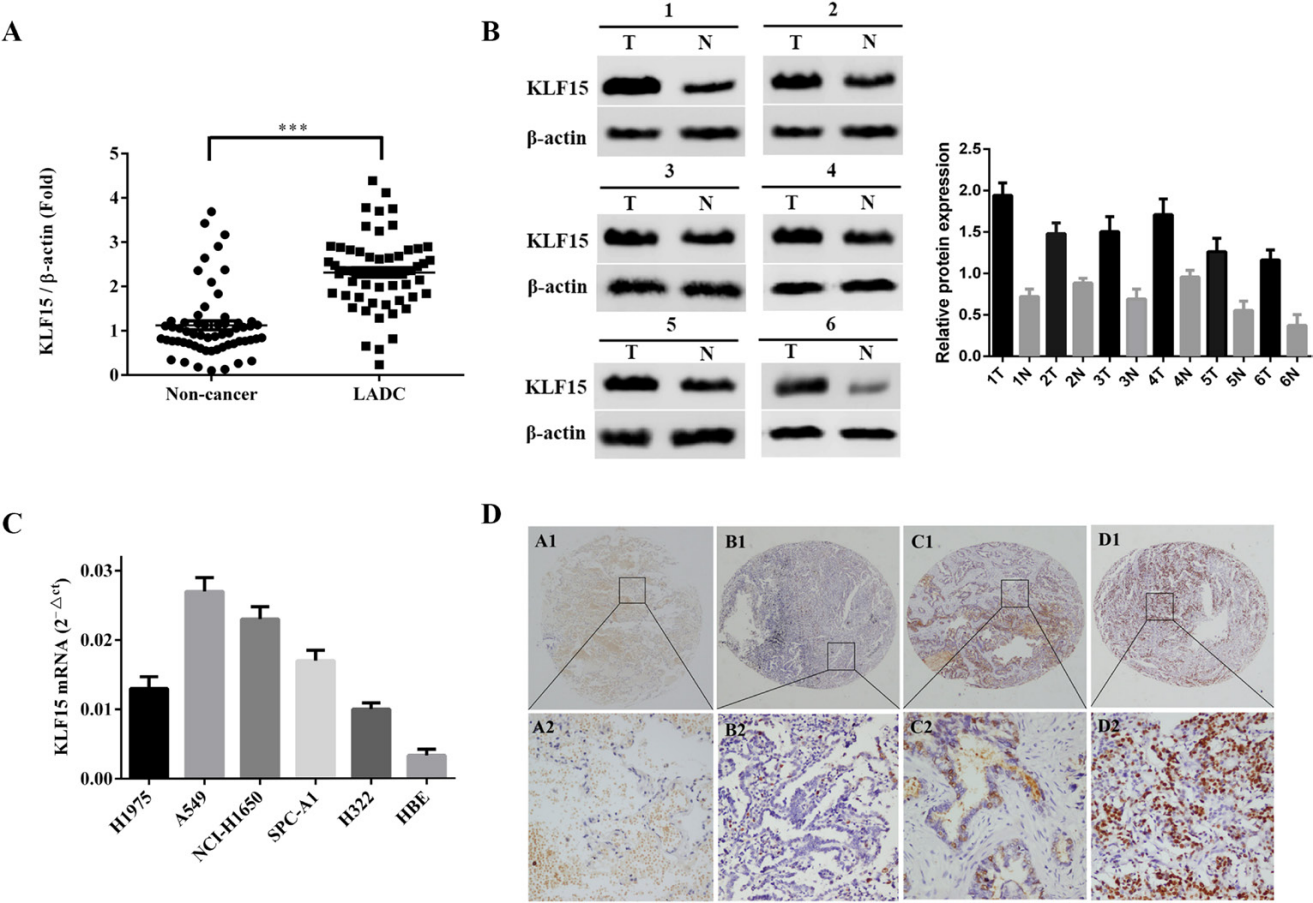
Upregulation of KLF15 in clinical specimens and LADC derived cell lines (**A**) The expression of KLF15 mRNA in 60 paired LADC and adjacent non-tumorous tissue specimens, as analyzed by qRT-PCR. (**B**) Representative results of the upregulation of KLF15in LADC specimens by Western Blotting analysis. (**C**) mRNA expression of KLF15 in five LADC cell lines and in normal human bronchial epithelial cells. (**D**) Representative results of the upregulation of KLF15 in LADC specimens as determined by immunohistochemistry analyses. ^*^P < 0.05, ^**^P < 0.01, ^***^P < 0.001.

**Figure 2:**
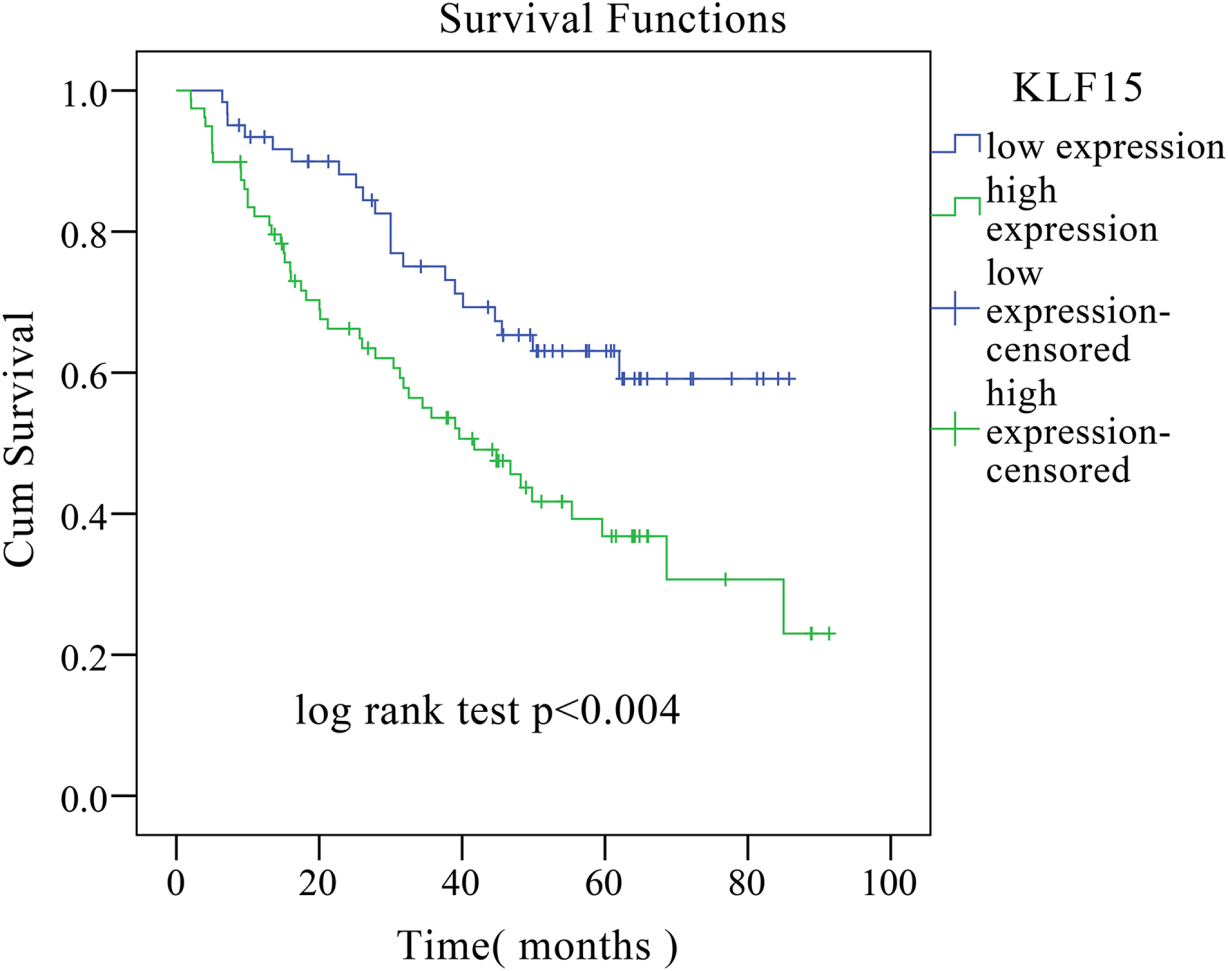
Kaplan–Meier overall survival curve of LADC patients correlated with KLF15 expression

To investigate the role of KLF15 in LADC, qRT-PCR was used to detect and quantify mRNA levels of KLF15 in 60 pairs of LADC and non-tumorous adjacent tissue specimens. The relative expression levels of KLF15 were markedly upregulated in tumor tissues compared with matched non-tumorous adjacent tissues (Figure 1A). The KLF15 protein expression levels of six typical pairs of LADC samples, as determined by Western Blotting, are shown in Figure 1B. We further investigated the expression of KLF15 in five LADC cell lines by RT-PCR. The results showed that KLF15 expression was overexpressed in H1975, A549, NCI-H1650, SPC-A1 and H322 cell lines compared with its expression in normal HBEs (Figure 1C). To further confirm the results obtained by qRT-PCR and Western blot, IHC analysis was employed to analyze the expression of KLF15 in LADC tissue specimens using TMAs. The results showed that seventy-nine out of 140 LADC samples (56.4%) exhibited high expression of KLF15 in LADC tissues, whilst only 12 out of 87 samples (13.8%) of the matched non-tumorous tissues showed high expression of KLF15. This indicated that the KLF15 expression level was upregulated in tumor tissues, and the expression in poorly differentiated tumor tissues was higher than that in highly differentiated (Figure 1D). We also investigated the correlation between KLF15 expression and certain pathological variables of the LADC patients, and found that significant association between high KLF15 expression levels were significantly positively correlated with both tumor TNM stage (*P* = 0.003) and tumor differentiation (*P* = 0.001) (Table 1). Kaplan–Meier analysis indicated that KLF15 overexpression was correlated with poor overall likelihood of survival in LADC patients. Multivariate Cox regression analyses further revealed that KLF15 was an independent prognostic marker for the overall survival time of LADC patients (*P* = 0.045) (Table 2 and Figure 2).

**Table 1:** Correlation of KLF15 expression in tumorous tissues with clinicopathologic characteristics in LADC patients

Clinicopathologic characteristics	*n*	KLF15			
		Low or no expression	High expression	Pearsonχ^2^	*P*
Total	140	61 (43.57)	79 (56.43)		
Gender				0.574	0.449
Male	39	15 (38.46)	24 (61.54)		
Female	101	46 (45.54)	55 (54.46)		
Age at diagnosis(years)				1.391	0.238
≤ 60	52	26 (50.00)	26 (50.00)		
> 60	88	35 (39.77)	53 (60.23)		
Smoking				0.433	0.805
No	66	28 (42.42)	38 (57.58)		
Yes	33	16 (48.48)	17 (51.52)		
Unknown	41	17 (41.46)	24 (58.54)		
Differentiation				17.456	0.001^*^
Low grade	51	14 (27.45)	37 (72.55)		
Middle grade	51	20 (39.22)	31 (60.78)		
High grade	38	27 (71.05)	11 (28.95)		
Primary tumor				0.233	0.630
T1+T2	49	20 (40.82)	29 (59.18)		
T3+T4	91	41 (45.05)	50 (54.95)		
Lymph node metastasis				0.163	0.922
No regional lymph node metastasis	77	34 (44.16)	43 (55.84)		
Ipsilateral peribronchial metastasis	39	16 (41.03)	23 (59.97)		
Mediastinal metastasis	24	11 (45.83)	13 (54.17)		
Stage Grouping with TNM				11.920	0.003^*^
Stage I	60	37 (61.67)	23 (38.33)		
Stage II	53	18 (33.96)	35 (66.04)		
Stage III+IV	27	8 (29.63)	19 (70.37)		

^*^ *P* < 0.05

**Table 2:** Univariate and multivariate analysis of the association of prognosis with clinicopathologic parameters and KLF15 expression in LADC patients

Characteristic	Univariate analysis			Multivariate analysis		
	HR	*P*	95%CI	HR	*P*	95%CI
KLF15 expression High vs Low	2.101	0.005*	1.251–3.527	1.757	0.045*	1.014–3.045
Gender	1.064	0.826	0.612–1.850			
Male vs Female						
Age (years)	0.896	0.658	0.549–1.460			
≤60 vs >60						
Smoking history	0.568	1.183	0.664–2.109			
Yes vs No						
Differentiation	1.471	0.016*	1.074–2.015	1.338	0.094	1.061–1.921
Low vs middle vs high grade						
Primary tumor	1.212	0.354	0.807–1.819			
T1 +T2 vs T3+T4						
Lympho node metastasis	1.437	0.015*	1.072–1.927	1.428	0.019*	1.061–1.921
N0 vs N1 vs N2						
TNM stage	1.352	0.057	0.991–1.845			
I vs II vs III+IV						

^*^ *P* < 0.05

### Knockdown of KLF15 suppresses cell growth *in vitro* and *in vivo*

**Figure 3:**
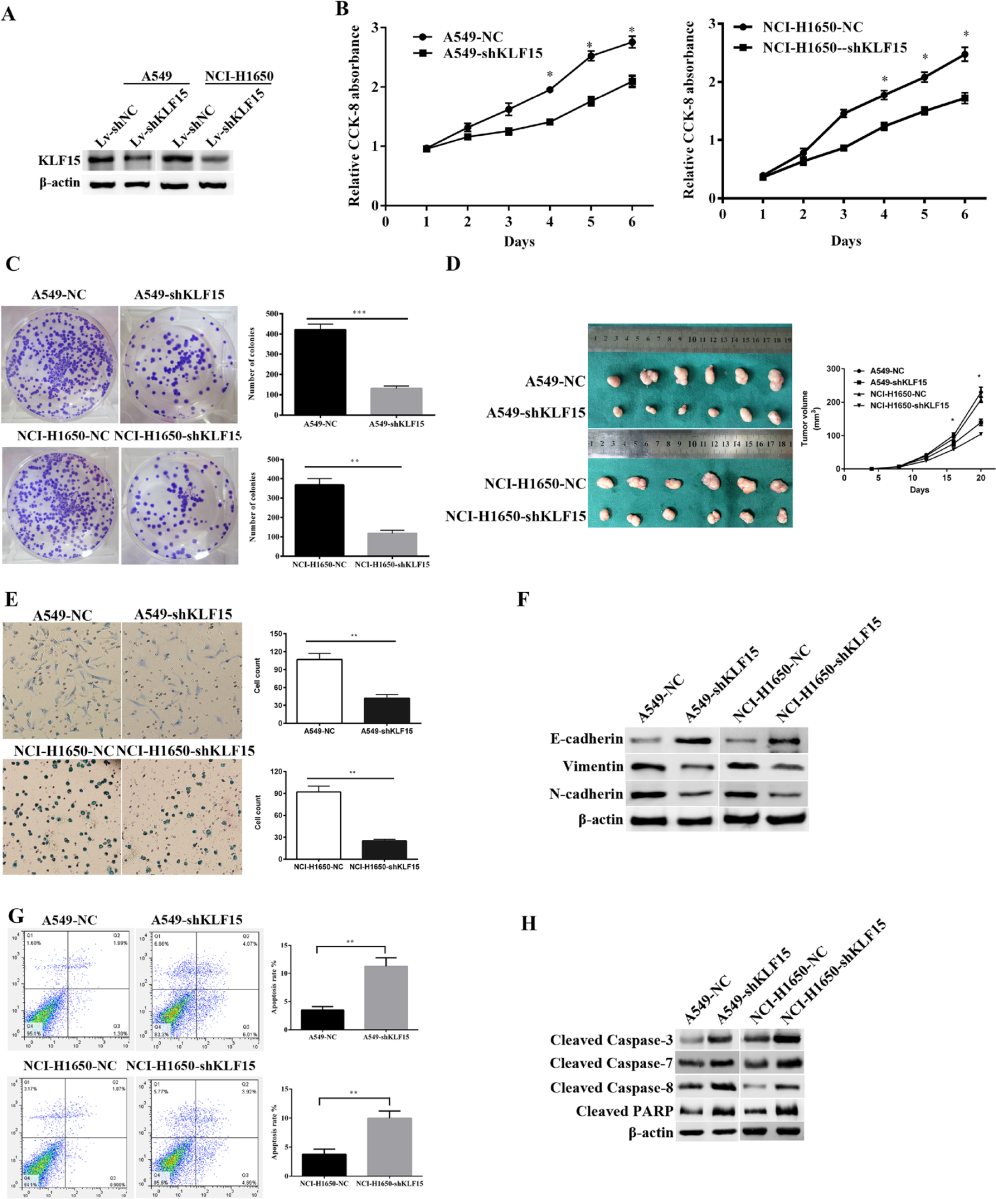
The effect of KLF15inLADC on cell proliferation, tumor growth, apoptosis and the expression of EMT related proteins (**A**) The expression of KLF15in KLF15-depletedA549 and NCI-H1650 cell lines. (**B**) Cellular proliferation of treated LADC cells as measured by CCK8 cell proliferation assay at various time points. (**C**) Proliferating colonies in incubated A549 and NCI-H1650 cells as determined by the colony formation assay. Statistical significance was determined on the basis of the numbers of identified colonies, as identified by light microscopy. The numbers of colonies are shown in the column charts. (**D**) LADC Tumor sizes in nude BALB/c male mice measured every fifth day after inoculation with KLF15 transfected or controlA549 and NCI-H1650 cells. (**E**) The metastatic condition of treated A549 and NCI-H1650 cells as measured by cell migration assays. (**F**) Western Blotting analysis of the expression levels of Vimentin, N-cadherin and E-cadherin in KLF15 transfected and control A549 and NCI-H1650 cells. (**G**) Cell apoptosis in KLF15 transfected and control A549 and NCI-H1650 cells, as determined by annexin V-FITC and PI staining using flow cytometry. The representative apoptosis pattern is shown, and the apoptotic cells are indicated in the UR and LR quadrants. (**H**) Knockdown of KLF15 in A549 and NCI-H1650 cells induced expression of the cleaved forms of caspase-3, caspase-7, caspase-8 and PARP, as determined by Western Blot analysis. All data shown are means ± SD. *P < 0.05. β-actin was used as the loading control.

We chose A549 and NCI-H1650 cell lines with high KLF15 expression to utilize *in vitro* knockdown experiments in order to analyze the potential oncogenic function of KLF15. Our results showed that the expression of KLF15 was significantly downregulated in A549-shKLF15 and NCI-H1650-shKLF15 cells compared with control cells, which was validated by Western Blotting analysis (Figure 3A). Furthermore, knockdown of KLF15 significantly suppressed cell growth in A549 and NCI-H1650 cell lines, compared with the control group (Figure 3B). As expected, the colony formation ability in KLF15-transfected A549 and NCI-H1650 cell lines were also found to be significantly reduced (Figure 3C). Collectively, these results indicate that KLF15 is vital in promoting LADC cell growth.

To examine the *in vivo* cell proliferative effects of KLF15 in LADC, the infected A549 and NCI-H1650 cell lines were subcutaneously injected into nude mice and the tumor volume and weight were evaluated. As shown in Figure 3D, downregulation of KLF15 in A549 and NCI-H1650 cell lines resulted in these cell lines exhibiting markedly slower growth rates compared with the comparator control groups (*P* < 0.05).

### Knockdown of KLF15 suppressed LADC cell migration

To further explore the potential effects of KLF15 on LADC cell metastasis, we performed a transwell migration assay. The results indicated that the downregulated expression of KLF15 significantly suppressed cell migratory capability in the A549-shKLF15 and NCI-H1650-shKLF15 cell lines in comparison with the comparator control groups (Figure 3E). Furthermore, we examined the expression levels of related epithelial-mesenchymal transition (EMT) protein markers, including E-cadherin, Vimentin and N-cadherin, in the A549 and NCI-H1650 cells transfected with shKLF15, by Western Blotting. Our results showed that the knockdown of KLF15 reduced the expression levels of N-cadherin and Vimentin while enhancing the expression of E-cadherin in the A549 and NCI-H1650 cells (Figure 3F). These results suggest that KLF15 promotes migratory abilities of LADC cells by modulating related EMT biomarkers.

### Silencing of KLF15 promotes LADC cell apoptosis

To identify the molecular mechanisms by which KLF15 may be involved in the proliferation and metastasis of LADC cells, we performed flow cytometry to investigate the effect of KLF15 on apoptosis. The results showed that the apoptotic indexes of KLF15-silenced cells were significantly lower than those of control cells, in both A549 and NCI-H1650 transfected cells (11.25% *vs.*3.50% for A549 cells(*P* < 0.01);9.98 % *vs.* 3.77 % for NCI-H1650 cells, (*P* < 0.01)) (Figure 3G). We also measured levels of a number of apoptosis related proteins and found that knockdown of KLF15 significantly upregulated the levels of cleaved caspase-3, caspase-7, caspase-8 and PARP in KLF15-transfected A549 and NCI-H1650 cells (Figure 3H). These data indicate that KLF15 promotes LADC cell progression by reducing cell apoptosis.

## DISCUSSION

The mechanisms of expression of specific implicated genes and of signal transduction pathways involved in the cellular invasion and migration processes of several different cancers have been the subject of research attention in several recent studies [18–22]. Whilst significant progress has been made in the treatment of LADC, high mortality rates in LADC patients remain. With the development of LADC research, an increasing number of potentially useful biomarkers are emerging. KLF15, as a member of the KLF family, has been linked to increased tumor growth in pancreatic, endometrial and breast cancers, likely the results of its role in promoting cell proliferation [16, 17]. However, the underlying functions of KLF15 have received less research attention in LADC. Against this background, the present study sought to reveal the role and mechanisms of KLF15 in LADC metastasis.

The results of the real-time RT-PCR and Western Blotting analyses demonstrated that there is significantly increased expression of KLF15 in human LADC tissues and cell lines compared with that in non-tumorous tissues. Following a more specific analysis of the clinicopathological characteristics of patients, we discovered that this elevated expression of KLF15 was closely related to the TNM stage of tumors, as well as to tumor differentiation and a poorer overall survival time in LADC patients. Cox proportional hazard regression analysis further identified KLF15 as an independent factor predicting poor prognosis, suggesting increased KLF15 expression could be a predictive marker of the development of LADC.

The confirmation of over-expression of KLF15 in LADC by the qRT-PCR, Western Blotting and IHC analyses presented an ideal model with which to study the role and molecular mechanisms of KLF15 in the pathology of LADC. As shown by the results of the CCK-8 and colony formation assays, the proliferation of LADC cells could be suppressed by silencing KLF15 expression *in vitro*, an effect that was validated *in vivo* via injection of transfected LADC cell lines in to nude mice. Additionally, trans-well migration assays revealed that the down regulation of KLF15 expression could inhibit migration of both A549 and NCI-H1650 cells, indicating thatKLF15 might go through genetic alteration related to metastasis in LADC cells. As a complex cellular process and the most frequent cause of death in LADC patients, metastasis involves the spread of tumor cells from a primary tumor to a secondary site within the body, and involves various complex molecular and cellular factors related to cell proliferation and migration, degradation of the basement membrane, invasion, adhesion and angiogenesis.

At the molecular level, cancer metastasis is caused by the acquisition of genetic and/or epigenetic alterations, along with the cooperation of stromal cells [23, 24]. Epithelial-mesenchymal transition (EMT) is a crucial step in tumor progression, playing a critical role in cancer invasion and metastasis. During the EMT process, the properties of epithelial cells are deprived and mesenchymal phenotypes are acquired. Higher expression of mesenchymal-related markers, such as Vimentin, and lower expression of epithelial-related markers, such as E-cadherin, are exhibited by mesenchymal phenotype cells [25, 26]. In the present study, based on the Western Blotting analysis of EMT related markers, it was found that knockdown of KLF15 induced EMT by reducing the levels of N-cadherin and Vimentin and by elevating expression of E-cadherin in the A549 and NCI-H1650 cells. These findings indicate that KLF15 regulated EMT in LADC cancer cells, leading to metastasis.

Apoptosis is a type of cell death, requiring a series of molecules including signaling molecules, enzymes, receptors, and gene regulating proteins, especially caspase family proteases [27, 28]. As a major mechanism in cancer development, the inhibition of apoptosis results in the expansion of neoplastic cells with deregulated proliferation and, ultimately, an accumulation of genetic instability and mutations [29]. Caspases constitute a family of end proteases which provide critical links in cell regulatory networks to regulate inflammation and cell death. The activation of apoptotic caspases leads to the inactivation or activation of substrates, and the occurrence of a cascade of signaling events that permits the controlled demolition of cellular components [30]. In the present study, cell apoptosis assays revealed that the growth-promotive effect of KLF15 was related to the inhibition of apoptosis, including the upregulation of the levels of cleaved caspase-3, caspase-7, caspase-8 and PARP. Therefore, impaired LADC cell growth and metastasis due to KLF15 silencing can be inferred by inactivation of the caspase-cascade signaling pathway that is responsible for KLF15 shRNA-mediated suppression of tumor cell proliferation, migration, invasion, and EMT.

Previous reports have demonstrated that KLF15 plays the role both in promoting cancer and suppressing cancer, then we conducted more than 3 times experiments to verify the role of KLF15 in LADC. The results all demonstrated that KLF15 played a vital role in promoting LADC cancer. The expression of KLF15, the different stage of cancer, the heterogeneity between tumor and normal or tumor tissue, different tumor microenvironment, and different signal pathways all may lead to the difference effect of KLF15 in different cancers. However, the current study is limited, the specific and in-depth study will be performed in the future.

In conclusion, we have demonstrated that the expression of KLF15 is abnormally high in LADC tissues and cells. Downregulating the expression of KLF15 had a significant inhibitory effect on the proliferation and migration of LADC cells. Further exploration revealed that KLF15 suppressed apoptosis in LADC cells to promote tumor growth. As a result, our findings suggest that KLF15 may play a vital role in LADC tumor cell survival, and that it has potential value as a therapeutic and preventive biomarker for LADC.

## MATERIALS AND METHODS

### LADC tissue samples

A total of 140 paired lung adenocarcinoma and adjacent normal lung tissue samples were obtained from patients with LADC at the Department of General Surgery, Affiliated Hospital of Nantong University, Jiangsu Province, China. All of these patients had undergone radical resection (including lymph-adenectomy) without prior chemotherapy or radiotherapy. Adjacent normal (non-tumorous) lung tissue samples were obtained from the lung that had undergone surgery. After resection, tissue samples were immediately stored in liquid nitrogen. The remainder of the LADC tissue specimens and cognate non-cancerous tissue specimens were fixed in formalin after surgery and embedded in paraffin for immunohistochemistry (IHC) analysis. The disease grade and histological type of all tissue samples were assessed and confirmed by two professional pathologists independently. All patients had provided informed consent prior to the start of the study, and the study had been approved by the Human Research Ethics Committee of Nantong University Affiliated Hospital, Jiangsu Province, China.

### LADC cell lines and transfection

Human lung adenocarcinoma cell lines (H1975, A549, NCI-H1650, SPC-A1 and H322) and normal human bronchial epithelial cells (HBEs) were purchased from the American Type Culture Collection (Manassas, Virginia, USA), and cultured in RPMI 1640 medium containing 10% fetal bovine serum, ampicillin and streptomycin, at 37°C in a humidified cell incubator with an atmosphere of 5% CO_2_. The A549 cells and NCI-H1650 cells were transfected with plasmids encoded with shRNA against KLF15, along with the empty vector controls. The shRNA targeting sequence for KLF15 was 5′-CTACCCTGGAGGAGATTGAAG-3′. The cDNA encoding full-length human KLF15 was cloned into a pCDH vector. The expression constructs were verified by DNA sequencing.

### RNA extraction and quantitative real-time polymerase chain reaction (qPCR) analysis

Total RNA was extracted from tissue samples and quantitative real-time PCR was performed based on previously described procedures [8]. The comparative Ct method was used to evaluate the expression levels of KLF15 mRNA in the tissue samples and cells of the different groups [11]. β-actin was used as the internal control. All experiments were performed in triplicate.

### Protein extraction and western blotting analysis

Protein extraction and Western Blotting were conducted based on previously described methods [31]. Rabbit anti-caspase-3, rabbit anti-caspase-7, rabbit anti-caspase-8, rabbit anti-Vimentin, rabbit anti-N-cadherin, and rabbit anti-E-cadherin antibodies were purchased from Cell Signalling Technology (Beverly, MA, USA). Rabbit anti-KLF15 and rabbit anti-GAPDH antibodies were purchased from Abcam (Cambridge, MA, USA). β-actin was used as the loading control. All experiments were performed in triplicate.

### Tissue microarray (TMA) construction and immunohistochemistry (IHC) analysis

LADC tissue specimens and matched normal (non-cancerous) tissue specimens (*n* = 140) were prepared and used for TMAs. We used a Tissue Microarray System (Quick-Ray, UT06, UNITMA, Korea) for the analysis. Core tissue biopsies (2 mm in diameter) from individual paraffin-embedded sections were arranged in recipient paraffin blocks. Sections (4 μm in thickness) of the TMA specimens were placed on super frost-charged glass microscope slides.

IHC analysis was carried out according to previously described methods [32]. The product of the intensity and percentage scores was used as the final KLF15 staining score. After calculation, we used ROC curve in SPSS to get a cut-off value of 192.5. The degree of KLF15 staining was quantified with a two-level grading system, the samples which got a score higher than the cut-off value were defined as “high expression (2–9)”, while the samples with a score lower than the cut-off value were defined as “low expression (0–1)”. The results of IHC staining were imaged using SPOT imaging software (Nikon) under microscope.

### Cell proliferation assay

Cells were plated at a density of 5 × 10^3^ cells per well in 96-well plates and incubated at 37°C in an atmosphere of 5% CO_2_, with three replicate wells per group. Subsequently, CCK-8 solution (Dojindo Laboratories, China) was added to each well for the indicated time periods. After 3 h of incubation, the optical density (OD) at a wavelength of 450 nm (OD450) was measured based on the manufacturer's instructions.

### Colony formation assay

A549 and NCI-H1650 cells were seeded in 6-well plates (100 cells/well). After 3 weeks culturing in culture medium containing 1,000 μg/ml G418, proliferating colonies were isolated and fixed with paraformaldehyde, stained with crystal violet, and then counted. All experiments were performed in triplicate.

### Tumorigenicity assay in nude mice

Male 5-week-old BALB/c nude mice were purchased from the Laboratory Animal Center, Nantong University, China. LADC cells (shNC or shKLF15 group) were subcutaneously inoculated into the flank of the mice (5×10^6^ cells/mouse, 6 mice per experimental group). Tumor size was monitored and measured every fifth day after inoculation. Tumor volume was calculated using the following formula: tumor volume (mm^3^) = (width^2^ × length)/2. Tumor weight was measured after about three weeks, following euthanasia of the experimental mice. The care of experimental animals was in accordance with the institutional animal care and use guidelines of Nantong University, China.

### Cell apoptosis analysis by flow cytometry

Annexin v-FITC/PI staining was used to measure live, apoptotic and necrotic cells. LADC cells were harvested and treated with a mixture of 100 μl Annexin v-FITC and PI [33]. Flow cytometry was then used to classify fluorescent cells. The number of apoptotic cells was analyzed by BD FACSuite software.

### Cell migration assay

Cell migration was examined using a BioCoat Matrigel Invasion Chambers equipped with 8-μm transwell filters (BD Bio-sciences). LADC cells (5 × 10^4^) were suspended in the upper chamber with serum-free medium, whilst medium containing 10% FBS was placed in the bottom chamber as a chemo attractant. After incubation for 24 h at 37°C all cells on the upper surface of the membrane were removed and stained with coomassie brilliant blue followed by manual counting under a light microscope in three randomly selected fields of view (Olympus, Tokyo, Japan). All procedures were performed in triplicate.

### Statistic analysis

Data were collected and analyzed using SPSS17.0 software and are expressed as means ± standard deviation (SD). Kaplan–Meier plots and log-rank tests were used for survival analysis. Univariate and multivariate Cox proportional hazard regression models were used to analyze independent prognostic factors. Differences between two groups were analyzed by two-tailed Student's *t*-tests. Categorical data were evaluated by the χ^2^ test. Probability values of *P* < 0.05 were considered statistically significant.
